# The impact of tumor budding and single-cell invasion on survival in patients with stage III/IV locally advanced oral squamous cell carcinoma- results from a prospective cohort study

**DOI:** 10.3389/fonc.2024.1404361

**Published:** 2024-04-29

**Authors:** Samer G. Hakim, Ubai Alsharif, Mohamed Falougy, Lars Tharun, Dirk Rades, Justus Jensen

**Affiliations:** ^1^ Department of Maxillofacial Surgery, University Hospital Schleswig-Holstein, Lübeck, Germany; ^2^ Department of Oral and Maxillofacial Surgery, Helios Medical Center, Schwerin, Germany; ^3^ Department of Oral and Maxillofacial Surgery, Dortmund General Hospital, Dortmund, Germany; ^4^ Faculty of Health, Witten/Herdecke University, Witten, Germany; ^5^ Department of Pathology, University Hospital Schleswig-Holstein, Lübeck, Germany; ^6^ Department of Radiation Oncology, University Hospital Schleswig-Holstein, Lübeck, Germany

**Keywords:** oral squamous cell carcinoma, tumor budding, risk factors, single-cell invasion, survival

## Abstract

**Introduction:**

Tumor budding (TB) refers to the presence of small clusters of tumor cells at the invasive front of a malignant tumor. Single tumor cell invasion (SCI) is an extreme variant of TB, in which individual loose tumor cells are present at the invasive front. Both TB and SCI are important histomorphologic risk factors postulated to indicate loss of cellular cohesion. In this study, we investigated the influence of TB and SCI on different survival outcomes in patients with locally advanced oral squamous cell carcinoma (OSCC).

**Methods:**

We included 129 patients with locally advanced OSCC (pT3-4) from a single-center, prospectively maintained cohort. We examined the association of TB and SCI with the presence of occult lymph node metastasis using a logistic regression model. Survival probabilities were estimated using the Kaplan-Meier method and cumulative incidence functions. The association of TB and SCI on overall survival (OS), oral cancer-specific survival (OCSS), and local recurrence-free survival (LRFS) was investigated using Cox’s proportional hazards regression models.

**Results:**

TB was detected in 98 (76%) of the tumors, while SCI was observed in 66 (51%) patients. There was a significant association between TB and the occurrence of occult lymph node metastasis (OR=3.33, CI: 1.21-10.0). On multivariate analysis, TB had no detectable impact on survival outcomes. However, SCI showed a higher risk for local recurrence (Hazards ratio (HR): 3.33, CI: 1.19 – 9.27).

**Discussion:**

This study demonstrates that TB and SCI in locally advanced OSCC function as an independent risk factor for occult lymph node metastases, as well as local recurrences. Both histomorphologic risk factors could serve as an additional parameter for stratifying therapy and escalating multimodal treatment approaches.

## 1 Introduction

Oral squamous cell carcinoma (OSCC) is the most common malignancy of the head and neck region, with more than 300,000 new cases and 145,000 cancer-related deaths yearly (1, 2).

Still characterized by a poor prognosis, promising prognostic markers are warranted to better stratify patients and treatment recommendations. The inherent morphologic features and infiltration pattern at the invasion front of OSCC may represent such a potential marker to estimate the survival outcomes in these patients.

For instance, the effect of Depth Of Infiltration (DOI) as a histologic feature on disease prognosis was evident in several studies, which led to the incorporation of DOI in the T category in the current 8^th^ edition of both UICC and AJCC TNM classifications for oral cancer current TNM classification (3).

This DOI-based upgrade subsequently led to therapy escalation in the sense of recommendation for adjuvant radiotherapy in patients with the new T3 category.

Another emerging and promising prognostic histomorphological marker for aggressive tumor invasion, metastasis, and poor prognosis is tumor budding (TB) and its extreme variant, single-cell invasion (SCI). TB is described as isolated tumor cells or in small clusters (<5 cells) present in the stroma, while SCI is defined as a single tumor cell invasion along the invasive margin of the tumor (4). Both TB and SCI are an expression of 2 distinct properties of malignancy: loss of cellular cohesion and active invasive movement, and both are morphologic correlates of the epithelial-mesenchymal transition (EMT) and were recently found to be associated with poor prognosis in tongue carcinoma (5, 6).

The role of TB has already been established as an independent prognostic factor in several cancer entities. However, very few studies highlighted its role in OSCC, only dealing with early-stage tumors and trying to stratify the outcome and therapy according to a semi-quantitative score comparable to that established for colorectal, breast, and lung cancer (7–9). Still, none has evaluated its impact on the survival outcomes in locally advanced III/IV stages of OSCC.

The most crucial aspect in addressing T3 and T4 tumors is the regular recommendation for postoperative adjuvant radiotherapy. The standard concurrent platin-based chemotherapy is mainly advised once additional risk factors are present (e.g., extranodal extension, involved margin … etc.). Tumor budding and increased depth of infiltration (DOI) are additional histologic risk factors that may influence this recommendation, encouraging therapy escalation.

Therefore, to evaluate this aspect, we encountered all patients with locally advanced stages of OSCC in a prospectively maintained single-center cohort, investigated the histologic risk markers DOI, TB, and SCI and analyzed their impact on the overall survival (OS), oral cancer-specific survival (OCSS), and locoregional disease-free survival (LRDFS) while adjusting to possible confounders such as resection margin (R-status) and nodal status (N-status).

## 2 Material and methods

### 2.1 Study population

**Figure 1:**
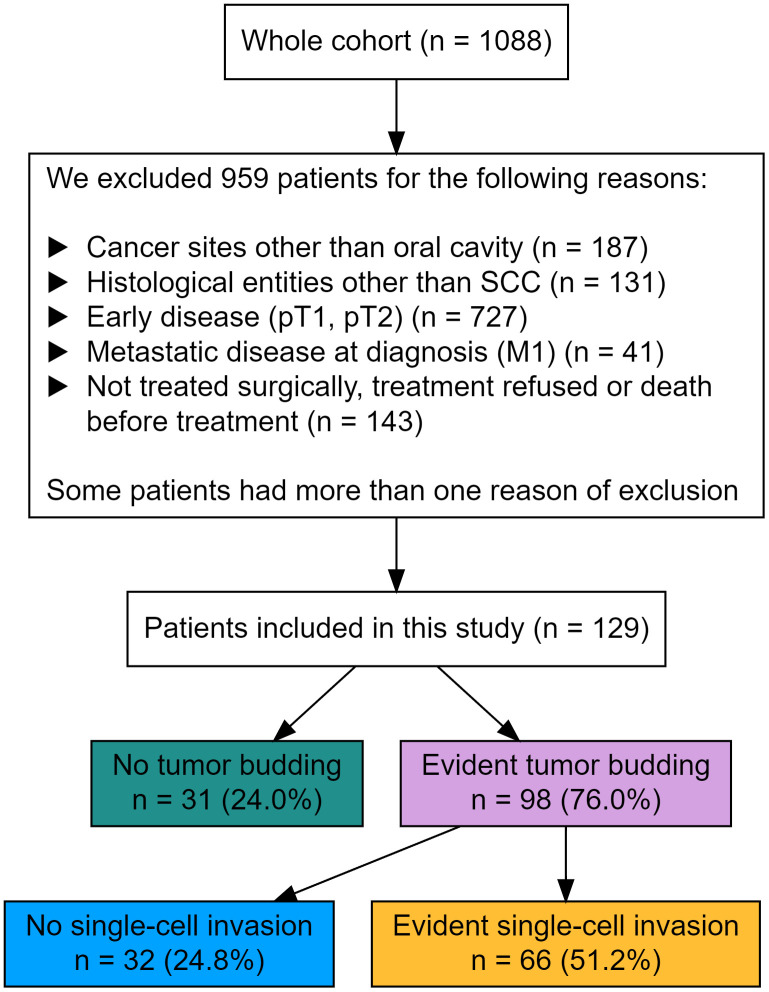
Study design, exclusion criteria, and distribution of groups.

All cases with a primary OSCC without distant metastasis from a cohort of 1088 patients were identified within the tumor data bank of the Department of Maxillofacial Surgery at the University Medical Centre of Lübeck, Germany. All patients underwent primary diagnostic investigation and therapy between 1996 and 2019. This study only included curatively treated patients with known T3/4 N0/+ and M0 status. Patients with distant metastasis, cancer of unknown primary, and a history of head and neck cancer with or without irradiation were excluded. **Figure 1** shows the criteria for exclusion.

The treatment decision and the decision for adjuvant radiochemotherapy (RCT) was made by the local head and neck multidisciplinary team. Adjuvant RCT was recommended for patients with T3/T4 and/or N+ tumors. Surgical treatment included radical tumor resection controlled by intraoperative frozen sections, uni- or bilateral selective or radical neck dissection, as well as individual, defect-based reconstruction, mostly using microvascular free flaps.

Patients’ clinical data were obtained from a prospectively maintained single-center cohort and included patients’ demographics, risk factors and clinical tumor characteristics. Treatment decisions were available at the baseline and at each follow-up. We used the updated version of the Charlson’s comorbidity index (CCI) to capture comorbidities and categorized CCI into no significant comorbidities (score=0), and at least one score point (CCI score ≥ 1) (10).

### 2.2 Histopathologic assessment

First, all histological sections were viewed using the light microscope, and a representative specimen with maximum tumor extension and relation to the healthy oral mucosa was selected for each patient. These were then scanned and digitized. The Ventana iScan HT scanner was used to visualize the slides (Ventana, Tuscon, AZ, USA). The QuPath (University of Edinburgh, UK) image analysis software was used for the digital evaluation of the slides.

Depth of Invasion (DOI), tumor budding (TB), and single-cell invasion (SCI) were determined as follows. Depth of invasion was measured from the basement membrane level of the cancer section to the deepest point of the invasive tumor in the stroma of the paraffin-embedded sections (11). The cutoff point to stratify the patients with OSCC into the low-risk and high-risk tumors was set at 10mm (12, 13).

Pathologists reported the shortest distance between the tumor mass and the specimen border histologically to evaluate the resection margin and stratified it as follows:

- Free margin (R0): Complete excision with a clear margin of ≥5 mm.
- Close margin (R0cm): Complete excision with a clear margin between 1-4 mm.
- High-risk margins: specimen with at least one involved margin, but all frozen sections are clear (R0hr).
- Incomplete excision: Frozen sections with microscopical tumor infiltration (R1) (14).

Tumor budding was defined as the presence of small cell nests with less than five tumor cells. It is assessed within the invasion front with a defined magnification. Magnification was set at 300x, and buds were numerically recorded within the 795 x 960 µm field of view. The presence of at least one bud in the invasion front justified the assignment to the TB group (15–18). Values were expressed in a dichotomous manner, which means TB ≥ 1 buds.

**Figure 2:**
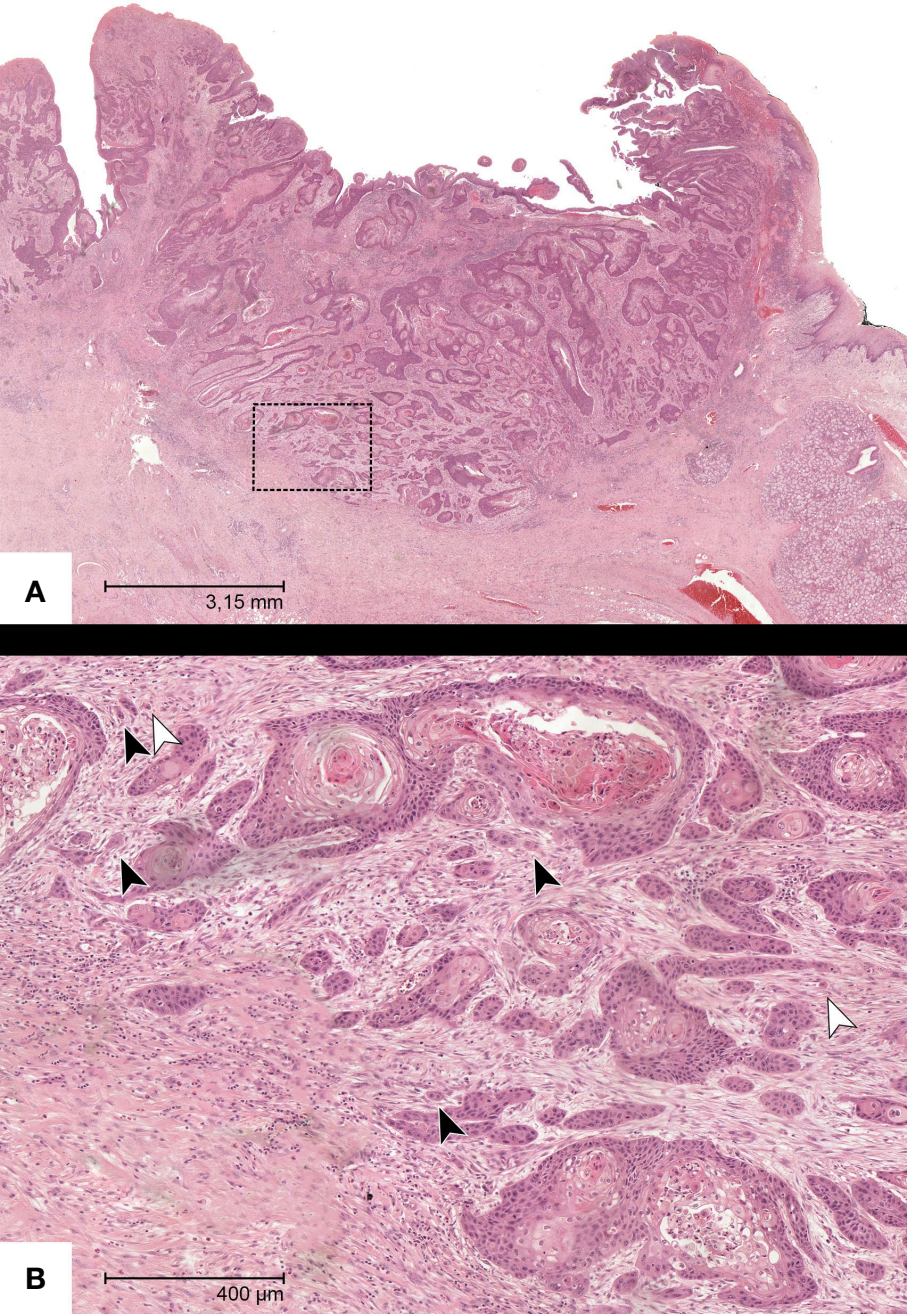
Representative HE-section of a specimen with OSCC in the overview **(A)**. **(B)** shows a magnification of the insert with manifest tumor budding (black arrows) and single-cell invasion (white arrows).

Single-cell invasion (SCI) was defined as isolated tumor cells segregated from the rest of the tumor (15, 16). Values were also recorded dichotomously. (**Figure 2**).

The remaining features were reported in the prospectively maintained cohort according to the 8^th^ edition of the TNM classification of malignant tumors (19).

All samples from the patient included were reviewed and re-evaluated critically by two investigators (L.T. and J.J.). Both were blinded to the clinical data at the time of the evaluation.

### 2.3 Follow-up and survival endpoints

All patients underwent routine recall and related clinical investigations every three months in the first two years and every six months for the last three years of the 5 years follow-up period. This follow-up included clinical, sonographic, and radiologic assessment and ended when patients either fulfilled 5 years of complete disease-free follow-up, decided to drop out from the regular aftercare, died, or the follow-up endpoint on the 31 of March 2019 was reached. Patients who were alive at the end of follow-up were censored. All survival durations were measured from the time point of the initial diagnosis. The endpoint of overall survival (OS) was death from any cause; the endpoint of oral cancer-specific survival (OCSS) was death from oral cancer; the endpoint of locoregional Disease-free survival (LRDFS) was the local or neck recurrence, while the endpoint of local recurrence-free survival (LRFS) was the occurrence of local recurrence only.

### 2.4 Statistical analysis

The baseline data was tabulated after calculating medians and interquartile range (IQR) for skewed variables. The median and 5-year survival probabilities, as well as 95% corresponding confidence intervals (CI), were estimated using the Kaplan-Meier method for survival analysis for OS and cumulative events for OCSS, LRFS, and LRRFS. The association between Tumor budding, DOI on one hand and resection margins or occult lymph node metastasis on the other hand was investigated using a logistic regression model after dichotomizing resection margins and occult lymph node metastasis. Here, we report the OR and its 95% confidence intervals. We estimated adjusted hazard ratios (HR) and corresponding 95% CI for TB and associated risk factors using Cox’s proportional hazards regression models. For OCSS, LRFS and LRDFS, we estimated adjusted cause-specific HRs in competing risks scenarios, which means that both outcomes had death from any cause other than oral cancer as a competing risk (20). All statistical analyses were performed using R Statistical Software (version 4.2.2; R Foundation for Statistical Computing, Vienna, Austria).

### 2.5 Ethics

All participants signed consent forms allowing their data to be collected and used anonymously for academic research on admission. The study was approved by the ethics review committee of the University of Lübeck.

## 3 Results

### 3.1 Patient demographics

A total of 129 patients were encountered and fulfilled the criteria for study inclusion. In the specimens of 31(24%) patients, no budding of tumor cells was detected. In 32 (25%) patients, tumor budding was observed alone, while in 66 (51%) patients, an associated single-cell invasion was assessed. SCI was never observed without budding.

Resection with clean margins was obtained in 27 patients (19% of patients with TB and 23% of those with TB and SCI). Specimens of 33 (26%) patients showed close margin resection (Rcm) (22% of patients with TB and 26% of those with TB and SCI), 48 (37%) specimens had only clean frozen sections, while the main specimen showed involved margins (R0hr) (50% of patients with TB and 35% of those with TB and SCI). Finally, 21 (16%) patients underwent R1 resection (9.4% of patients with TB and 17% of those with TB and SCI).

Based on the related literature, the cutoff for DOI was set at 10 mm (21, 22). In 48% of patients, DOI amounted to more than 10 mm (55% of patients with TB and 53% of those with TB and SCI), while in 52% of all cases, DOI was lower than10 mm (45% of patients with TB and 47% of those with TB and SCI).

All baseline characteristics are given in **Table 1**, stratified by the TB and SCI status.

**Table 1:** Baseline characteristics and disease recurrence stratified by tumor budding status and single-cell invasion.

Variable	Overall N = 129^1^	No budding N=31 (24%)^2^	Budding without SCI N=32 (25%)^2^	Budding with SCI N=66 (51%)^2^
**Median age (IQR)**	62 (55-71)	66 (55-72)	64 (56-73)	60 (53-69)
Gender				
* Male*	87 (67%)	24 (77%)	22 (69%)	41 (62%)
* Female*	42 (33%)	7 (23%)	10 (31%)	25 (38%)
CCI score				
* 0*	88 (68%)	19 (61%)	25 (78%)	44 (67%)
* 1 ≤*	41 (32%)	12 (39%)	7 (22%)	22 (33%)
Smoking				
* Never*	24 (19%)	10 (33%)	6 (19%)	8 (12%)
* Former or current*	103 (81%)	20 (67%)	25 (81%)	58 (88%)
* Missing*	2	1	1	0
Alcohol consumption				
* None or moderate*	42 (35%)	14 (50%)	10 (34%)	18 (29%)
* Excessive*	77 (65%)	14 (50%)	19 (66%)	44 (71%)
* Missing*	10	3	3	4
Subsite				
* Floor of mouth*	48 (37%)	11 (35%)	9 (28%)	28 (42%)
* Anterior tongue*	17 (13%)	3 (9.7%)	3 (9.4%)	11 (17%)
* Gum*	40 (31%)	7 (23%)	14 (44%)	19 (29%)
* Cheek, Vestibule, retromolar*	11 (8.5%)	6 (19%)	2 (6.3%)	3 (4.5%)
* Palate*	13 (10%)	4 (13%)	4 (13%)	5 (7.6%)
Pathological tumor size (pT)				
* T3*	50 (39%)	13 (42%)	12 (38%)	25 (38%)
* T4*	79 (61%)	18 (58%)	20 (63%)	41 (62%)
Nodal disease (pN)				
* N0*	69 (53%)	23 (74%)	15 (47%)	31 (47%)
* N1*	16 (12%)	3 (9.7%)	4 (13%)	9 (14%)
* N2a/b*	24 (19%)	3 (9.7%)	9 (28%)	12 (18%)
* N2c/3*	20 (16%)	2 (6.5%)	4 (13%)	14 (21%)
Grade of differentiation				
* Well*	7 (5.4%)	4 (13%)	2 (6.3%)	1 (1.5%)
* Moderate*	88 (68%)	22 (71%)	20 (63%)	46 (70%)
* Poor*	34 (26%)	5 (16%)	10 (31%)	19 (29%)
Resection margins				
* R0*	27 (21%)	6 (19%)	6 (19%)	15 (23%)
* R0cm*	33 (26%)	9 (29%)	7 (22%)	17 (26%)
* R0hr*	48 (37%)	9 (29%)	16 (50%)	23 (35%)
* R1*	21 (16%)	7 (23%)	3 (9.4%)	11 (17%)
**Number of buds**	4.0 (1.0-10.0)	-	3.0 (2.0-4.0)	9.5 (6.0-13.8)
Depth of invasion (mm)				
*<10*	67(52%)	22(71%)	14 (45%)	31 (47%)
*10<*	61 (48%)	9 (29%)	17 (55%)	35 (53%)
*Missing*	1	0	1	0
Lymphatic invasion				
Therapy				
* Surgery only*	38 (29%)	11 (35%)	7 (22%)	20 (30%)
* Adjuvant radiotherapy*	59 (46%)	13 (42%)	14 (44%)	32 (48%)
* Adjuvant radiochemotherapy*	32 (25%)	7 (23%)	11 (34%)	14 (21%)
Local recurrence in 5yr				
* Censored*	93 (72%)	25 (81%)	25 (78%)	43 (65%)
* Local recurrence*	36 (28%)	6 (19%)	7 (22%)	23 (35%)
Regional recurrence in 5yr				
* Censored*	112 (87%)	30 (97%)	27 (84%)	55 (83%)
* Regional recurrence*	17 (13%)	1 (3.2%)	5 (16%)	11 (17%)
Locoregional recurrence in 5yr				
* Censored*	89 (69%)	24 (77%)	23 (72%)	42 (64%)
* Locoregional recurrence*	40 (31%)	7 (23%)	9 (28%)	24 (36%)
Any disease recurrence in 5yr				
* Censored*	77 (60%)	19 (61%)	22 (69%)	36 (55%)
*Any disease recurrence*	52 (40%)	12 (39%)	10 (31%)	30 (45%)
Death from any cause				
*Alive or censored*	46 (36%)	9 (29%)	14 (44%)	23 (35%)
*Dead*	83 (64%)	22 (71%)	18 (56%)	43 (65%)
Cause of death				
* Alive or censored*	46 (36%)	9 (29%)	14 (44%)	23 (35%)
* Death from oral cancer*	42 (33%)	10 (32%)	10 (31%)	22 (33%)
* Death from other causes*	29 (22%)	9 (29%)	6 (19%)	14 (21%)
* Unknown cause of death*	12 (9.3%)	3 (9.7%)	2 (6.3%)	7 (11%)

^1^Median (25%-75%); n (%).

^2^Continous variables are presented using the median and inter-quartile range (25% - 75%).

### 3.2 Distribution of buds and single-cell invasion within the tumor specimens

**Figure 3:**
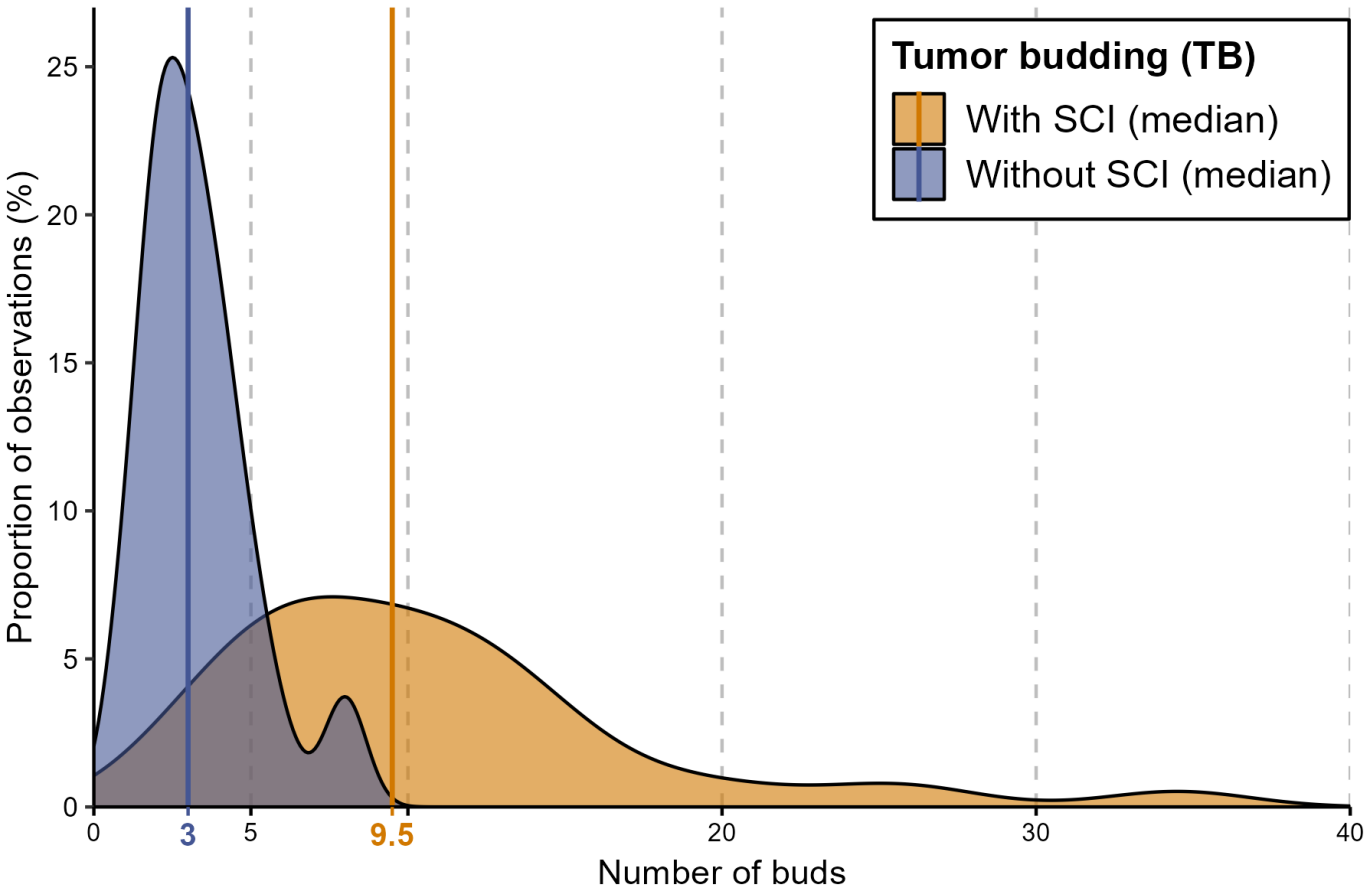
A density diagram showing the distribution of the number of buds in patients with TB stratified by SCI. This graph shows the strong correlation between a higher number of buds and the presence of SCI. The solid lines represent the median values of the corresponding groups. The group with no evidence of TB is not illustrated.

Descriptive analysis was performed to investigate the distribution of TB and SCI within the cohort. The median number of buds was 4 buds, while the mean reached 6.3. **Figure 3** shows the median number of buds within each group stratified by SCI.

The single-cell invasion was evident in the specimens of 66 patients (51%) correlated with the increased number of buds. In most patients with more than 5 buds (57 patients, 90%) single-cell invasion was evident, while SCI was assessed in only 9 patients (26%) with less than 5 buds. In other words, the higher the number of buds in the histological section, the higher the probability of including SCI.

### 3.3 Association of tumor budding with resection margins and occult lymph node metastasis

A correlation analysis was performed to investigate any potential interference between tumor budding and single-cell invasion on one side and the ability to achieve a clear resection margin and the occurrence of lymph node metastasis on the other side. Interestingly, neither of the mentioned factors was associated with unsafe resection margins. However, a strong association was seen between tumor budding and the occurrence of occult lymph node metastasis at the time of diagnosis (OR=3.33 CI: 1.21-10.0, p=0.02) (**Table 2**).

**Table 2:** The association of tumor budding with resection margins and occult lymph node metastasis (LNM). TB but not SCI tripled the risk of occult LNM.

	Resection margins (R0/R0cm vs. R0hr/R1)			Occult lymph node metastasis (pN0 vs. pN+)		
Variable	OR^1^	95% CI^1^	p-value	OR^1^	95% CI^1^	p-value
Tumor budding						
*No budding*	—	—		—	—	
* Budding without SCI*	1.57	0.51-4.95	0.4	**3.27**	**1.02-11.3**	**0.05**
* Budding with SCI*	0.87	0.33-2.29	0.8	**3.33**	**1.21-10.0**	**0.02**

^1^OR, Odds Ratio; CI, Confidence Interval.Significant values are provided in bold font.

### 3.4 Impact of TB and SCI on the survival outcomes

The follow-up ranged from 4 months to 12 years among survivors, with a median OS time of 20 (15–43) months in the whole cohort. In a cumulative follow-up duration of 326 person-years, 83 patients died, resulting in a death rate of 25.2 deaths per 100 person-years.

Within the 5 years of follow-up, 83 (64%) patients died, 42 (33%) patients died due to oral cancer, and 29 (22%) died from other causes. The cause of death was unknown in 12 (9.3%) patients. Additionally, 36 (28%) patients developed local recurrences and 17 (13%) suffered from regional recurrence.

**Figure 4:**
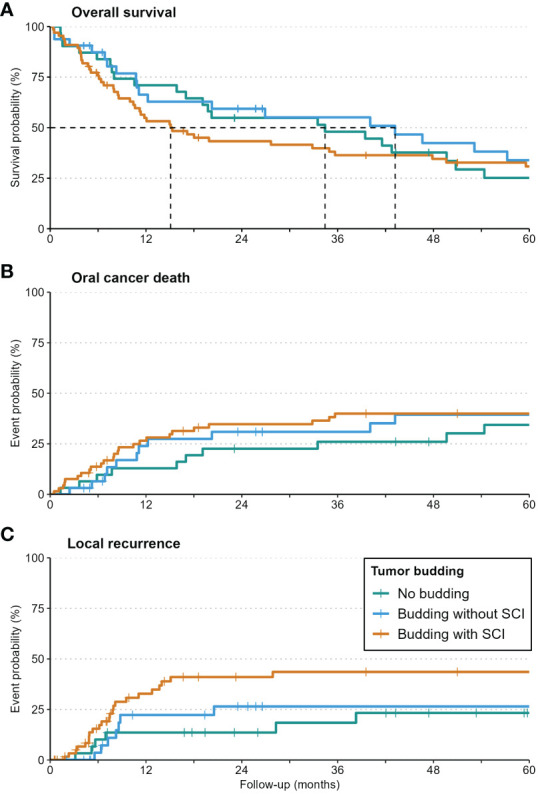
Kaplan-Meier-curves for Overall Survival **(A)**, Oral Cancer Death **(B)**, and local recurrence **(C)** in patients with no budding, budding with/without single-cell invasion. Local recurrence was increased significantly in patients with single-cell invasion.

At 5 years, a clear trend to local and locoregional recurrence was observed in patients with tumor budding (26% and 33%, respectively) and especially in patients with single-cell invasion (40% and 45%, respectively) in relation to patients without budding (**Table 3**, **Figure 4**).

**Table 3:** Observed 5-year probabilities and 95% CI of death and other survival outcomes in the cohort.

	Median survival (months)	Death by any cause	Death by oral cancer	Local recurrence	Locoregional recurrence
**Overall**	20 (15-43)	70% (60%-77%)	38% (30%-48%)	34% (26%-45%)	37% (29%-48%)
Tumor budding					
*No budding*	34 (17-54)	75% (52%-87%)	34% (21%-57%)	23% (11%-48%)	26% (14%-51%)
*Budding without SCI*	43 (12-76)	66% (42%-80%)	39% (25%-63%)	26% (14%-50%)	33% (19%-57%)
*Budding* *with SCI*	15 (11-48)	69% (55%-79%)	40% (29%-54%)	44% (32%-60%)	45% (33%-61%)

On multivariate analysis, and as expected, a significantly increased hazard ratio for all investigated survival outcomes was assessed in patients with primarily involved lymph nodes (N+ status) (HR: 2.05 – 8.65). HR for local recurrence was also elevated significantly in patients with incomplete resection (R1) or high-risk margins (R0hr) (HR: 4.66 and 4.93, respectively; both with p<0.05).

While budding alone had no detectable impact on any survival outcomes, budding with single-cell invasion increased the risk of local recurrence significantly (HR: 3.33, p=0.021). Detailed values are given in **Table 4**.

**Table 4:** multivariate analysis for the different endpoints of the study.

	OS			OCSS			LRFS			LRDFS		
Variable^1^	HR^2^	95% CI^2^	p-value	HR^2^	95% CI^2^	p-value	HR^2^	95% CI^2^	p-value	HR^2^	95% CI^2^	p-value
**Age**	**1.04**	**1.02-1.06**	**<0.001**									
Gender												
*Male*	—	—										
*Female*	**0.50**	**0.29-0.86**	**0.011**									
Depth of invasion (mm)												
*<10*	—	—		—	—		—	—		—	—	
*10<*	1.22	0.76-1.96	0.4	0.82	0.54-1.25	0.4	0.67	0.30-1.51	0.3	1.12	0.51-2.47	0.8
Nodal disease (pN)												
*N0*	—	—		—	—					—	—	
*N1*	**2.05**	**1.02-4.12**	**0.044**	**3.29**	**1.58-6.86**	**0.002**				**4.96**	**1.69-14.6**	**0.004**
*N2a/b*	1.56	0.79-3.09	0.2	**2.49**	**1.13-5.50**	**0.024**				**3.57**	**1.13-11.3**	**0.030**
*N2c/3*	**5.22**	**2.60-10.5**	**<0.001**	**2.93**	**1.45-5.92**	**0.003**				**8.65**	**2.92-25.6**	**<0.001**
Resection margins												
*R0*	—	—		—	—		—	—		—	—	
*R0cm*	1.64	0.82-3.26	0.2	1.27	0.46-3.53	0.6	3.52	0.90-13.7	0.070	2.29	0.59-8.97	0.2
*R0hr*	1.37	0.69-2.72	0.4	1.33	0.53-3.34	0.5	**4.93**	**1.44-16.9**	**0.011**	2.62	0.76-9.05	0.13
*R1*	**3.32**	**1.55-7.12**	**0.002**	1.12	0.48-2.61	0.8	**4.66**	**1.11-19.6**	**0.036**	3.40	0.73-15.9	0.12
Tumor budding												
*No budding*	—	—		—	—		—	—		—	—	
*Budding without SCI*	0.91	0.45-1.83	0.8	0.74	0.35-1.58	0.4	1.04	0.30-3.64	>0.9	0.57	0.14-2.28	0.4
*Budding with SCI*	1.22	0.69-2.17	0.5	0.77	0.41-1.43	0.4	**3.33**	**1.19-9.27**	**0.021**	1.60	0.54-4.72	0.4

(overall survival (OS), oral cancer-specific survival (OCSS), local recurrence-free survival (LRFS), and locoregional disease-free survival (LRDFS).Significant values are provided in bold font.

## 4 Discussion

Tumor budding (TB) and its extreme variant, single-cell invasion (SCI) – a pendant of the high-risk WPOI - are considered histomorphological markers for loss of cellular adhesion and are closely associated with epithelial-mesenchymal transition (EMT), the hallmark of invasion and metastasis (5, 17).

This histologic feature at the invasion front has thoroughly been investigated and established as a risk factor in colorectal cancer (23, 24). Although both colorectal and oral cancer involve the gastrointestinal tract and, therefore, tumor budding may appear relevant for the survival outcome OSCC as well, some crucial differences might have to be considered critically. For instance, in view of histopathology, colorectal cancer is an adenocarcinoma, mostly induced by malignant transformation of an adenoma (adenoma-carcinoma-sequence). In contrast, oral cancer is a squamous cell carcinoma and is induced by local noxae (alcohol and smoking), HPV infection and chronic inflammation (25). Therefore, transferring the impact of tumor budding on the survival outcomes from colorectal cancer into OSCC requires reliable data and solid evidence.

The current study was thus carried out to assess the prognostic significance of TB and SCI in locally advanced OSCC (stage III/IV), considering the confounders/biasing factors, such as N-status, R-status, DOI, and resection margin.

Recent studies showed a correlation of TB – evaluated as a dichotomous or semi-quantitative variable – with the potential of locoregional metastasis in OSCC (26–28). We assessed similar findings in the investigated cohort. However, the occurrence of occult lymph node metastasis and subsequent locoregional recurrence was only observed in specimens with both TB and SCI. Although the underlying hypothesis of such an association may appear logical, the interval between the initial diagnosis and the manifestation of lymph node metastasis is very variable, mostly censored, and causality is hard to prove. In other words, a tumor detected at an initial stage without evident lymph node involvement may also show TB at the invasion front and vice versa.

While the number of buds is recommended for the grading and classifying colorectal cancer patients, the Worst Pattern of Invasion (WPOI) has drawn more attention to risk classification in oral cancer. High-risk WPOI is marked by small tumor islands <15 cells and satellite tumor(s) located at least 1 mm from the main tumor or the nearest satellite (17, 29, 30). This histologic feature corresponds with the term “Single-Cell Invasion (SCI)” applied in the present study. With DOI, a risk score has been postulated by Almangush et al. based on budding (cutoff = 5 buds) and WPOI to escalate therapy in high-risk classified T1/2 oral tongue cancer. The cutoff of DOI used to stratify the patients into the low-risk and high-risk tumors in that study was set at 4 mm (22, 31, 32).

In the current investigation, however, we included T3/4 tumors that require adjuvant treatment per se. Thus, many confounding risk factors at primary diagnosis may be diminished and censored by the therapeutic effect of the following radiation. This aspect draws special attention when stratifying and assigning these patients to later adjuvant treatment.

The cutoff used in the present study was set at 10 mm to stratify for poor outcomes considering the subsequent treatment (radio- or radiochemotherapy). Therefore, the apparent discrepancy between our results and those of the literature given DOI-related stage and prognosis can be explained by the endpoint of either investigation. While the meta-analysis by Huang et al. aimed at detecting a threshold of tumor thickness/DOI for primary lymph node invasion (22), the endpoint applied in the present study was its impact on the survival outcomes, including local recurrence and disease-free survival.

DOI exceeding 10 mm brought no additional risk for any survival outcome within the investigated cohort. The absence of the effect of DOI on the survival outcome may be attributed to and explained by two possible hypotheses.

First, DOI may represent a marginal or dependent risk factor per se correlating with another factor, such as TB. These results would be concordant with recent studies that confirmed the correlation of TB with DOI (27, 33).

An alternative hypothesis may indicate the positive impact of adjuvant therapy in diminishing the difference in the DOI in both groups (10 mm <DOI< 10 mm). Studies addressing this aspect are very limited since most research groups have focused on early-stage OSCC to determine if a subgroup of these patients may profit from a therapy escalation (e.g., radio(chemo)therapy), which is regularly not recommended in T1/2 N0 tumors (34). Nevertheless, this is a new aspect to be addressed in future clinical investigations.

Studies evaluating the predictive and prognostic performance of TB in locally advanced OSCC are inhomogeneous in view of the included tumor stages, mostly investigated the correlation between TB and DOI or TB with lymph node metastasis and/or extranodal extension in early-stage disease, and rarely used multivariate analysis considering confounding risk factors (35–39).

As far as we know, the present study is the first to address only patients with locally advanced T3/4 N0-N+ tumors. Further, we used a new cutoff of TB to stratify the patients into low- and high-risk groups. Any TB manifestation was considered full-risk, and the patient was assigned to the TB group. This strict classification pattern is novel and has not been applied in previous studies yet (34). The findings shown when investigating the association with LNM indicate the crucial impact of this inherent histologic risk factor on the potential involvement of cervical lymph nodes in this patient group. Simultaneously, however, we observed a diminishing of this effect after adjuvant treatment in the long term since it didn’t influence locoregional-free survival. Similar results were reported by Bjerkli et al. and Manjula et al. (40) (41) once multivariate analysis was applied.

Regarding survival outcomes, TB alone was not a significant prognostic marker in the current investigation. SCI, in contrast, influenced the local recurrence rates, and its presence reduced the local recurrence-free survival in the cohort. This effect can be explained by the failure to achieve a clear margin in the surgical specimens, a feature observed obviously in the multivariate analysis. Boxberg et al. showed comparable findings in the survival analysis for oropharyngeal and laryngeal OSCC but failed to show any impact on the hazard ratio to develop local or loco-regional recurrence (16). A recently published study encountered similar results in the univariate analysis for single-cell invasion; however, it failed to confirm this in the multivariate Cox regression survival analyses (42).

Some limitations in the present study must be mentioned. While an experienced pathologist reviewed the histopathology material, section preparation improved over time, and the evaluation was optimized by immunohistology. This is especially true and important for assessing single-cell invasion, which can be performed more precisely using cytokeratin staining, especially when using automated evaluation. However, TB and SCI have been evaluated routinely by HE-staining, and the available data on the survival effect are based on this technique. The recommended staining by the ITBCC is the HE one and the vast majority of related studies were carried out using HE-staining. To provide a valid comparison HE-based evaluation by an experienced pathologist has been favored in the present study.

It also has to be noticed that the status of budding and SCI may change depending on the resection site and the evaluated section, and therefore, a hidden bias may appear, especially in large tumors with complex anatomical orientation.

Our study has several strengths and weaknesses. Including a homogenous group of patients with advanced stage III/IV disease is a strength and a limitation at the same time. Local cancer recurrence is the most immediate consequence of treatment failure of TB and SCI that we examined in our study, while death from oral cancer and overall mortality are merely following consequences. Independent of those three factors, this cohort of patients has by nature a high risk of local and regional disease recurrence and, consequently, a high rate of oral cancer death and overall mortality. As such, the fact that we detected an association of SCI with local recurrence but not for mortality is probably due to a high overall mortality rate in the cohort.

In addition, some treatment factors can modify the effect of the investigated histologic factors. Post-operative radiotherapy (PORT), for example, is usually recommended in patients with advanced OSCC. A modification of the effect of DOI and TB on the examined survival outcomes is very probable. Logically, PORT would weaken the measured effects of DOI, TB, and SCI and reduce the risk of local recurrence, diminishing the effect on overall and disease-specific mortality. Also, a higher risk of local recurrences might occur, but this does not mean those recurrences are not salvageable. A higher success rate of recurrence salvage surgery would also minimize the influence on overall and disease-specific mortality. Addressing the interactions of PORT and salvage surgery was beyond the scope of this research but may represent a subject of future studies.

The present study has a prospective nature. The patients’ follow-up was centralized in the study site, and the vital status of patients was acquired from the state’s cancer registry, allowing for optimal assessment of the disease recurrence and overall mortality. Together with the sound study design, addressing potential biases and competing survival risks, the current investigation provides solid data for upcoming clinical studies on this topic.

## 5 Conclusion

In conclusion, the aggravation of tumor budding in the sense of SCI may provide a novel diagnostic and prognostic biomarker, as well as a potential therapeutic tool for patients with locally advanced OSCC. Our results show that TB may be observed alone at the invasion front of locally advanced OSCC (stage II/IV), but SCI (WPOI grade 5) was always associated with TB within the investigated cohort.

In the present study, TB was associated with occult LNM. SCI did not increase the risk significantly. This indicates a higher risk for regional metastasis at diagnosis and before treatment. SCI increases the risk of local recurrence.

As closely addressed in recent studies and supported in the present one, especially for locally advanced OSCC, tumor budding and single-cell invasion are promising tissue-based biomarkers for prognosis and should be reported routinely. With prospective follow-up data, solid evidence can be provided to include TB and SCI in decision-making and the therapy guidelines for patients with oral cancer. Currently, both markers may be considered in surgical planning to extend neck dissection and escalate postoperative adjuvant therapy to include concurrent chemotherapy and/or immune therapy.

## Data availability statement

The datasets presented in this article are not readily available because the regulations of our local ethic committee do not allow data sharing. Requests to access the datasets should be directed to samer.hakim@uni-luebeck.de.

## Ethics statement

The studies involving humans were approved by Ethics Committee of the University of Luebeck in Germany. The studies were conducted in accordance with the local legislation and institutional requirements. Written informed consent for participation was not required from the participants or the participants’ legal guardians/next of kin because a retrospective analysis was conducted.

## Author contributions

SH: Conceptualization, Project administration, Supervision, Writing – original draft, Writing – review & editing. UA: Data curation, Formal analysis, Methodology, Visualization, Writing – original draft, Writing – review & editing. MF: Software, Writing – review & editing. LT: Data curation, Methodology, Writing – review & editing. DR: Data curation, Writing – review & editing. JJ: Data curation, Methodology, Visualization, Writing – original draft, Writing – review & editing.

## References

[1] Sung H Ferlay J Siegel RL Laversanne M Soerjomataram I Jemal A. Global cancer statistics 2020: GLOBOCAN estimates of incidence and mortality worldwide for 36 cancers in 185 countries. CA Cancer J Clin. (2021) 71:209–49. doi: 10.3322/caac.21660 33538338

[2] GBD 2019 Lip, Oral, and Pharyngeal Cancer Collaborators. The global, regional, and national burden of adult lip, oral, and pharyngeal cancer in 204 countries and territories: A systematic analysis for the global burden of disease study 2019. JAMA Oncol. (2023) 9:1401–16. doi: 10.1001/jamaoncol.2023.2960 PMC10485745 37676656

[3] Struckmeier AK Eichhorn P Agaimy A Buchbender M Moest T Lutz R. Comparison of the 7th and revised 8th UICC editions (2020) for oral squamous cell carcinoma: How does the reclassification impact staging and survival? Virchows Archh. (2024). doi: 10.1007/s00428-023-03727-y PMC11186894 38191928

[4] Togni L Caponio VCA Zerman N Troiano G Zhurakivska K Lo Muzio L. The emerging impact of tumor budding in oral squamous cell carcinoma: main issues and clinical relevance of a new prognostic marker. Cancers (Basel). (2022) 14. doi: 10.3390/cancers14153571 PMC9332070 35892830

[5] Wang C Huang H Huang Z Wang A Chen X Huang L. Tumor budding correlates with poor prognosis and epithelial-mesenchymal transition in tongue squamous cell carcinoma. J Oral Pathol Med. (2011) 40:545–51. doi: 10.1111/jop.2011.40.issue-7 PMC3135705 21481005

[6] Hakim SG Taubitz C Hoppe S Steller D Rades D Ribbat-Idel J. Prognostic impact of the loss of E-cadherin and *de novo* expression of N-cadherin at the invasive front of primary and recurrent oral squamous cell carcinoma. Front Oncol. (2023) 13:1151879. doi: 10.3389/fonc.2023.1151879 37265789 PMC10231494

[7] Rogers AC Winter DC Heeney A Gibbons D Lugli A Puppa G. Systematic review and meta-analysis of the impact of tumor budding in colorectal cancer. Br J Cancer. (2016) 115:831–40. doi: 10.1038/bjc.2016.274 PMC5046217 27599041

[8] Lloyd AJ Ryan EJ Boland MR Elwahab SA Malone C Sweeney KJ. The histopathological and molecular features of breast carcinoma with tumor budding-a systematic review and meta-analysis. Breast Cancer Res Treat. (2020) 183:503–14. doi: 10.1007/s10549-020-05810-3 32710280

[9] Thakur N Ailia MJ Chong Y Shin OR Yim K. Tumor budding as a marker for poor prognosis and epithelial-mesenchymal transition in lung cancer: A systematic review and meta-analysis. Front Oncol. (2022) 12:828999. doi: 10.3389/fonc.2022.828999 35719992 PMC9201279

[10] Quan H Li B Couris CM Fushimi K Graham P Hider P. Updating and validating the Charlson comorbidity index and score for risk adjustment in hospital discharge abstracts using data from 6 countries. Am J Epidemiol. (2011) 173:676–82. doi: 10.1093/aje/kwq433 21330339

[11] Jerjes W Upile T Petrie A Riskalla A Hamdoon Z Vourvachis M. Clinicopathological parameters, recurrence, locoregional and distant metastasis in 115 T1-T2 oral squamous cell carcinoma patients. Head Neck Oncol. (2010) 2:9. doi: 10.1186/1758-3284-2-9 20406474 PMC2882907

[12] Ebrahimi A Gil Z Amit M Yen TC Liao CT Chaturvedi P. Depth of invasion alone as an indication for postoperative radiotherapy in small oral squamous cell carcinomas: An International Collaborative Study. Head Neck. (2019) 41:1935–42. doi: 10.1002/hed.25633 PMC6563806 30801885

[13] Alterio D D’Urso P Volpe S Tagliabue M De Berardinis R Augugliaro M. The impact of post-operative radiotherapy in early stage (pT1-pT2N0M0) oral tongue squamous cell carcinoma in era of DOI. Cancers (Basel). (2021) 13. doi: 10.3390/cancers13194851 PMC8507768 34638335

[14] Hakim SG von Bialy R Falougy M Steller D Tharun L Rades D. Impact of stratified resection margin classification on local tumor control and survival in patients with oral squamous cell carcinoma. J Surg Oncol. (2021) 124:1284–95. doi: 10.1002/jso.26655 34416792

[15] Boxberg M Bollwein C Jöhrens K Kuhn PH Haller B Steiger K. Novel prognostic histopathological grading system in oral squamous cell carcinoma based on tumor budding and cell nest size shows high interobserver and intraobserver concordance. J Clin Pathol. (2019) 72:285–94. doi: 10.1136/jclinpath-2018-205454 30530818

[16] Boxberg M Kuhn PH Reiser M Erb A Steiger K Pickhard A. Tumor budding and cell nest size are highly prognostic in laryngeal and hypopharyngeal squamous cell carcinoma: further evidence for a unified histopathologic grading system for squamous cell carcinomas of the upper aerodigestive tract. Am J Surg Pathol. (2019) 43:303–13. doi: 10.1097/PAS.0000000000001178 30475254

[17] Xu B Salama AM Valero C Yuan A Khimraj A Saliba M. The prognostic role of histologic grade, worst pattern of invasion, and tumor budding in early oral tongue squamous cell carcinoma: a comparative study. Virchows Arch. (2021) 479:597–606. doi: 10.1007/s00428-021-03063-z 33661329 PMC8417140

[18] Lugli A Kirsch R Ajioka Y Bosman F Cathomas G Dawson H. Recommendations for reporting tumor budding in colorectal cancer based on the International Tumor Budding Consensus Conference (ITBCC) 2016. Mod Pathol. (2017) 30:1299–311. doi: 10.1038/modpathol.2017.46 28548122

[19] Brierley J Gospodarowicz MK Wittekind C. TNM classification of Malignant tumors. Eight edition. Chichester, West Sussex, UK; Hoboken, NJ: John Wiley & Sons, Inc (2017).

[20] Kalbfleisch JD Prentice RL. The Statistical Analysis of Failure Time Data. Wiley (2011).

[21] Pentenero M Gandolfo S Carrozzo M. Importance of tumor thickness and depth of invasion in nodal involvement and prognosis of oral squamous cell carcinoma: a review of the literature. Head Neck. (2005) 27:1080–91. doi: 10.1002/hed.20275 16240329

[22] Huang SH Hwang D Lockwood G Goldstein DP O’Sullivan B. Predictive value of tumor thickness for cervical lymph-node involvement in squamous cell carcinoma of the oral cavity: a meta-analysis of reported studies. Cancer. (2009) 115:1489–97. doi: 10.1002/cncr.24161 19197973

[23] Dawson H Galuppini F Trager P Berger MD Studer P Brugger L. Validation of the International Tumor Budding Consensus Conference 2016 recommendations on tumor budding in stage I-IV colorectal cancer. Hum Pathol. (2019) 85:145–51. doi: 10.1016/j.humpath.2018.10.023 30428391

[24] Chen K Collins G Wang H Toh JWT. Pathological features and prognostication in colorectal cancer. Curr Oncol. (2021) 28:5356–83. doi: 10.3390/curroncol28060447 PMC8700531 34940086

[25] Espressivo A Pan ZS Usher-Smith JA Harrison H. Risk prediction models for oral cancer: A systematic review. Cancers (Basel). (2024) 16. doi: 10.3390/cancers16030617 PMC10854942 38339366

[26] Shimizu S Miyazaki A Sonoda T Koike K Ogi K Kobayashi JI. Tumor budding is an independent prognostic marker in early stage oral squamous cell carcinoma: With special reference to the mode of invasion and worst pattern of invasion. PloS One. (2018) 13:e0195451. doi: 10.1371/journal.pone.0195451 29672550 PMC5909609

[27] Almangush A Heikkinen I Makitie AA Coletta RD Laara E Leivo I. Prognostic biomarkers for oral tongue squamous cell carcinoma: a systematic review and meta-analysis. Br J Cancer. (2017) 117:856–66. doi: 10.1038/bjc.2017.244 PMC5589992 28751758

[28] Hurnik P Reznarova J Chyra Z Motyka O Putnova BM Cermakova Z. Enhancing oral squamous cell carcinoma prediction: the prognostic power of the worst pattern of invasion and the limited impact of molecular resection margins. Front Oncol. (2023) 13:1287650. doi: 10.3389/fonc.2023.1287650 38188288 PMC10766711

[29] Brandwein-Gensler M Teixeira MS Lewis CM Lee B Rolnitzky L Hille JJ. Oral squamous cell carcinoma: histologic risk assessment, but not margin status, is strongly predictive of local disease-free and overall survival. Am J Surg Pathol. (2005) 29:167–78. doi: 10.1097/01.pas.0000149687.90710.21 15644773

[30] Binmadi NO Mohamed YA. Impact of worst pattern of invasion on prognosis of oral squamous cell carcinoma: a systematic review and meta-analysis. J Int Med Res. (2023) 51:3000605231206260. doi: 10.1177/03000605231206260 37871621 PMC10594968

[31] Berdugo J Thompson LDR Purgina B Sturgis CD Tuluc M Seethala R. Measuring depth of invasion in early squamous cell carcinoma of the oral tongue: positive deep margin, extratumoral perineural invasion, and other challenges. Head Neck Pathol. (2019) 13:154–61. doi: 10.1007/s12105-018-0925-3 PMC6514023 29700721

[32] Dirven R Ebrahimi A Moeckelmann N Palme CE Gupta R Clark J. Tumor thickness versus depth of invasion - Analysis of the 8th edition American Joint Committee on Cancer Staging for oral cancer. Oral Oncol. (2017) 74:30–3. doi: 10.1016/j.oraloncology.2017.09.007 29103748

[33] Niranjan KC Raj M Hallikeri K. Prognostic evaluation of tumor budding in oral squamous cell carcinoma: Evidenced by CD44 expression as a cancer stem cell marker. Pathol Res Pract. (2023) 251:154883. doi: 10.1016/j.prp.2023.154883 37898041

[34] Zanoletti E Daloiso A Nicole L Cazzador D Mondello T Franz L. Tumor budding to investigate local invasion, metastasis, and prognosis of head and neck carcinoma: A systematic review. Head Neck. (2024) 46:651–71. doi: 10.1002/hed.27583 38013617

[35] Angadi PV Patil PV Hallikeri K Mallapur MD Hallikerimath S Kale AD. Tumor budding is an independent prognostic factor for prediction of lymph node metastasis in oral squamous cell carcinoma. Int J Surg Pathol. (2015) 23:102–10. doi: 10.1177/1066896914565022 25559273

[36] Hamada M Ebihara Y Nagata K Yano M Kogashiwa Y Nakahira M. Podoplanin is an efficient predictor of neck lymph node metastasis in tongue squamous cell carcinoma with low tumor budding grade. Oncol Lett. (2020) 19:2602–8. doi: 10.3892/ol PMC7068445 32218810

[37] de Assis EM Rodrigues M Vieira JC Pascoaloti MIM Junior HM Souto GR. Lymphatic vascular density, the expression of podoplanin and tumor budding in oral squamous cell carcinoma. Head Neck Pathol. (2023) 17:371–82. doi: 10.1007/s12105-022-01511-z PMC10293516 36480090

[38] Ho YY Wu TY Cheng HC Yang CC Wu CH. The significance of tumor budding in oral cancer survival and its relevance to the eighth edition of the American Joint Committee on Cancer staging system. Head Neck. (2019) 41:2991–3001. doi: 10.1002/hed.25780 31012518

[39] Noda Y Ishida M Ueno Y Fujisawa T Iwai H Tsuta K. Novel pathological predictive factors for extranodal extension in oral squamous cell carcinoma: a retrospective cohort study based on tumor budding, desmoplastic reaction, tumor-infiltrating lymphocytes, and depth of invasion. BMC Cancer. (2022) 22:402. doi: 10.1186/s12885-022-09393-8 35418058 PMC9006434

[40] Manjula BV Augustine S Selvam S Mohan AM. Prognostic and predictive factors in gingivo buccal complex squamous cell carcinoma: role of tumor budding and pattern of invasion. Indian J Otolaryngol Head Neck Surg. (2015) 67:98–104. doi: 10.1007/s12070-014-0787-2 25621262 PMC4298608

[41] Bjerkli IH Laurvik H Nginamau ES Soland TM Costea D Hov H. Tumor budding score predicts lymph node status in oral tongue squamous cell carcinoma and should be included in the pathology report. PloS One. (2020) 15:e0239783. doi: 10.1371/journal.pone.0239783 32976535 PMC7518591

[42] Tan A Taskin T. Tumor budding should be in oral cavity cancer reporting: A retrospective cohort study based on tumor microenvironment. Cancers (Basel). (2023) 15. doi: 10.3390/cancers15153905 PMC10416929 37568721

